# Transfer and Persistence of a Multi-Drug Resistance Plasmid *in situ* of the Infant Gut Microbiota in the Absence of Antibiotic Treatment

**DOI:** 10.3389/fmicb.2017.01852

**Published:** 2017-09-26

**Authors:** Heidi Gumpert, Jessica Z. Kubicek-Sutherland, Andreas Porse, Nahid Karami, Christian Munck, Marius Linkevicius, Ingegerd Adlerberth, Agnes E. Wold, Dan I. Andersson, Morten O. A. Sommer

**Affiliations:** ^1^Department of Systems Biology, Technical University of Denmark, Lyngby, Denmark; ^2^Department of Clinical Microbiology, Hvidovre Hospital, University of Copenhagen, Hvidovre, Denmark; ^3^Department of Medical Biochemistry and Microbiology, Uppsala University, Uppsala, Sweden; ^4^The Novo Nordisk Foundation Center for Biosustainability, Technical University of Denmark, Lyngby, Denmark; ^5^Department of Infectious Diseases, Institute of Biomedicine, Sahlgrenska Academy, University of Gothenburg, Gothenburg, Sweden

**Keywords:** *Escherichia coli*, horizontal gene transfer, infant gut, genome dynamics, plasmid transfer, *in vivo* fitness, mouse models, antibiotic resistance

## Abstract

The microbial ecosystem residing in the human gut is believed to play an important role in horizontal exchange of virulence and antibiotic resistance genes that threatens human health. While the diversity of gut-microorganisms and their genetic content has been studied extensively, high-resolution insight into the plasticity, and selective forces shaping individual genomes is scarce. In a longitudinal study, we followed the dynamics of co-existing *Escherichia coli* lineages in an infant not receiving antibiotics. Using whole genome sequencing, we observed large genomic deletions, bacteriophage infections, as well as the loss and acquisition of plasmids in these lineages during their colonization of the human gut. In particular, we captured the exchange of multidrug resistance genes, and identified a clinically relevant conjugative plasmid mediating the transfer. This resistant transconjugant lineage was maintained for months, demonstrating that antibiotic resistance genes can disseminate and persist in the gut microbiome; even in absence of antibiotic selection. Furthermore, through *in vivo* competition assays, we suggest that the resistant transconjugant can persist through a fitness advantage in the mouse gut in spite of a fitness cost *in vitro*. Our findings highlight the dynamic nature of the human gut microbiota and provide the first genomic description of antibiotic resistance gene transfer between bacteria in the unperturbed human gut. These results exemplify that conjugative plasmids, harboring resistance determinants, can transfer and persists in the gut in the absence of antibiotic treatment.

## Introduction

The evolution of multidrug resistant bacteria through horizontal gene transfer (HGT) is resulting in human pathogens that are no longer amenable to antibiotic therapy (Davies and Davies, 2010). It is believed that antibiotic resistance genes are frequently exchanged between bacteria within the human microbiome, where the intestinal bacterial community in particular is considered a hub for HGT (Liu et al., 2012). Transfer of antibiotic resistance genes within the gut microbiota is believed to happen primarily via conjugative plasmids and has been demonstrated to occur in both animals (McConnell et al., 1991; Schjørring et al., 2008), and humans (Lester et al., 2006; Trobos et al., 2009). Due to the low transfer frequency, and initial instability of plasmids in the absence of selection, previous studies have utilized experimental set-ups where the host was inoculated with a high number of bacteria, with subsequent monitoring to detect if the antibiotic resistance genes had been transferred from the donor strain (McConnell et al., 1991; Lester et al., 2006; Schjørring et al., 2008; Trobos et al., 2009).

We and others have documented the transfer of antibiotic resistance genes amongst naturally occurring bacteria in the human gut microbiota, and these reports describe changes in the antibiotic resistance profiles of strains collected from patients undergoing antibiotic treatment (Bidet et al., 2005; Karami et al., 2007; Conlan et al., 2014, 2016; Porse et al., 2017). Additionally, a retrospective study examining *Bacteroides* isolates, collected over a period of 40 years, demonstrated that extensive resistance gene exchange occurred between species of *Bacteroides* and other genera in the human colon (Shoemaker et al., 2001). Yet, while the unperturbed gut microbiome has been the subject of numerous metagenomic studies (Balzola et al., 2010; Huttenhower et al., 2012; Forslund et al., 2013), including the construction of complete genomes of various species and strains from metagenomic data (Sharon et al., 2013), the use of metagenomics is not well-suited to detect HGT events due to difficulties in associating mobile genetic elements with individual genomes.

To investigate the dynamics of horizontal gene exchange between *Escherichia coli* of the unperturbed gut microbiota, we use whole genome sequencing to characterize co-existing *E. coli* lineages isolated over the first year of an infant's life. Observing the transfer and enrichment of a conjugative antibiotic resistance plasmid, along with subsequent genomic events, in the absence of antibiotic treatment, we performed *in vivo* fitness assays indicating that this resistance plasmid is maintained in a gut environment despite being costly *in vitro*.

## Materials and methods

### Strain isolation and population counts

Fecal samples were obtained from an infant enrolled in the ALLERGYFLORA study (Nowrouzian et al., 2003). A sample of the rectal flora was obtained using a cotton-tipped swab at 3 days after birth. The infant's parents collected fecal samples at 1, 2, and 4 weeks, and 2, 6, and 12 months of age. Samples were plated on Drigalski agar plates for the isolation of *Enterobacteriaceae* with a detection limit of 10^2.5^ CFU/g fecal matter. Each morphotype was enumerated separately, and strain identities of the enumerated morphotypes were confirmed using random amplified polymorphic DNA (RAPD) typing (Nowrouzian et al., 2003). Initial confirmation of the RAPD-typing was confirmed by pulsed-field gel electrophoresis (PFGE). Isolated strains were subjected to complete serotyping (O:K:H) (Statens Serum Institute, Copenhagen, Denmark).

From the 5 sampling times positive for *E. coli*, a total of 13 isolates were selected and stored for further analysis.

### Antibiotic susceptibility and minimum inhibitory concentration (MIC) determination

All isolates were tested for their susceptibility to the following antibiotics using the disc diffusion method (Oxoid, Sweden): ampicillin, amoxicillin/clavulanic acid, piperacillin, mecillinam, cefadroxil, ceftazidime, cefuroxime, cefoxitin, chloramphenicol, gentamicin, tobramycin, streptomycin, nitrofurantoin, nalidixic acid, tetracycline, trimethoprim, and sulphonamide. From the saved isolates, the exact MICs of one isolate per lineage per sampling point were determined using the broth dilution method (Table S1; Wiegand et al., 2008).

### Genome sequencing

Genomic DNA from each of the 13 isolates was obtained using the UltraClean® Microbial DNA Isolation Kit (Mobio Laboratories, Inc.). Sequencing was performed by Partners HealthCare Center for Personalized Genetic Medicine (Massachusetts, USA) or at the Novo Nordisk Foundation Centre for Biosustainability (Lyngby, Denmark).

### Sequence analysis

Reads from each isolate were assembled using Velvet (Zerbino and Birney, 2008). Contigs with <500 bp were filtered and corrected by aligning reads using Bowtie2 (version 2.1.0) (Langmead et al., 2009). Single-nucleotide polymorphisms (SNPs) were called using SAMtools (version 0.1.19) (Li et al., 2009), and edited using custom biopython scripts (Cock et al., 2009). Contigs were annotated using the RAST server (Aziz et al., 2008). SAMtools were also used to determine the number of SNPs between isolates, where identified variants had a phred quality score of at least 50 and >90% of the high-quality reads as the variant. The assemblies from the following isolates where used as references for SNP-calling: lineage A 2w_2_, lineage B 2m_2_ and lineage C 12m_2_. SNPs occurring in short homologous regions after genomic deletions or acquisitions were also filtered. BEDtools (Quinlan and Hall, 2010) was used to calculate read coverage across genomes and thus identify acquired or deleted genomic information. MUMmer was used to align sequences (Khan et al., 2009). Multi-locus sequence type (MLST) groups were determined using the database hosted at http://mlst.warwick.ac.uk/mlst/dbs/Ecoli (Wirth et al., 2006).

### Phage identification

The PHAST phage search tool server (Zhou et al., 2011) was used to identify possible intact phages in the contigs. In addition, BLAST was used to identify similar previously described phages. Phage integration sites were determined by aligning contigs containing the flanking regions of the phage to an earlier isolate not containing the prophage.

### Plasmid analysis

The PlasmidFinder web-service (http://cge.cbs.dtu.dk/services/PlasmidFinder) was used to identify replicons in the assembled contigs and classify plasmids into incompatibility groups (Carattoli et al., 2014). Plasmid diagrams depicting read coverage were drawn in R via custom scripts, and plasmid ring diagrams were drawn using BLAST Ring Image Generator (BRIG; Alikhan et al., 2011) with the “-task megablast” option to BLAST. Additionally, contigs belonging to plasmids (that had a copy number greater than one) were identified based on their relative abundance to the genome via BEDTools (Quinlan and Hall, 2010).

### Genomic deletion verification by PCR

Based on the alignment of contigs to the genome of CFT 073 (NC_004431), flanking primers were designed to show that the deletion in lineage A was a chromosomal excision. In addition to show contiguity prior to the deletion, controls were included to show the occurrence of the deletion only in the lineage A isolate sampled at 6 months.

### *In vitro* conjugation assay

To test the ability of Lineage B to transfer the pHUSEC41-1-like plasmid to the plasmid free Lineage A, outgrown overnight cultures of two lineages were mixed equally and incubated for 12 h. Incubations were done at 37°C on a solid agar surface as well as in liquid cultures without shaking. Mating cultures were plated on LB containing chloramphenicol and ampicillin to select for transconjugants.

### *In vitro* competition experiments to assess the fitness costs of the pHUSEC41-1-resembling plasmid

To assess fitness cost, pairwise growth competition experiments in Davis minimal medium with 25 mg/mL glucose (DM25) were performed using isolates of lineage A sampled at 2 weeks and 2 months, respectively, the latter which had acquired the plasmid closely resembling pHUSEC41-1 (Künne et al., 2012). The experiment was performed as previously described (Enne et al., 2005), but in brief, the two isolates were grown overnight in nutrient broth, and then inoculated into DM25 at a dilution of 1:10^4^ and grown for 24 h. The cultures were then mixed together in a ratio of 1:1, and then diluted 1:100 into fresh DM25. The serial passage step was continued for 6 days, corresponding to ~60 generations of competition. After initially mixing the two cultures together, and after each 24 h period, the cultures were diluted appropriately and 100 μL were added to Iso-Sensitest plates (Oxoid, Sweden) in triplicate, with and without 50 mg/L of ampicillin. Colonies were counted after over-night incubation at 37°C, where the mean number of colonies on ampicillin plates was subtracted from the plates without ampicillin to determine the mean number of colonies lacking the pHUSEC41-1-like plasmids. Six replicates of the fitness experiment were conducted.

### *In vivo* competitive fitness assays

Isolates used in the competitive fitness studies were tagged with chloramphenicol (Cam^R^) and kanamycin (Kan^R^) resistance markers, *cat* and *aph(3*′*)-II* genes, respectively, amplified from cloning vectors of the pZ vector system (Lutz and Bujard, 1997): lineage A 2w_1_—Cam^R^, 2m—Kan^R^, 6m_1_—Cam^R^, and lineage C at 12m_1_—Kan^R^. The markers were inserted into the chromosomal *araB* gene of the lineage A and B strains using the Lambda Red recombineering system of pTKRED (Kuhlman and Cox, 2010). The following regions of homology were used for insertions into *araB*: 5′- GTAGCGAGGTTAAGATCGGTAATCACCCCTTTCAGGCGTTGGTTAGCGTT-3′ and 5′-GCCTAACGCACTGGTAAAAGTTATCGGTACTTCCACCTGCGACATTCTGA-3′.

Previous studies have shown that the inactivation of *araB* is fitness neutral in a murine model and that the Cam^R^ and Kan^R^ markers do not significantly affect the growth of *E. coli* (Chen et al., 2013; Linkevicius et al., 2016).

Female BALB/c mice (5–6 weeks old) were used in all *in vivo* experiments (Charles River Laboratories, distributed by Scanbur). All mice were pre-treated orally with streptomycin as described previously (Lasaro et al., 2014). Briefly streptomycin sulfate salt (Sigma-Aldrich) was added to the drinking water at 5 g/L, along with 5 g/L of glucose to enhance taste, for 72 h followed by 24 h of fresh water (no drug or glucose) to allow the streptomycin to be cleared from the animal's system prior to inoculation. No streptomycin was administered during the course of infection. Ten mice were administered 100 μL containing a 1:1 *E. coli* mixture by oral gavage of the examined strains. Feces were homogenized in PBS, serially diluted, and equal amounts were plated on LA-Cam (25 μg/ml chloramphenicol, selecting for the chromosomal marker), LA-Kan (50 μg/ml kanamycin, selecting for the chromosomal marker) and either LA-Kan, Amp or LA-Cam, Amp (50 μg/ml kanamycin or 25 μg/ml chloramphenicol, and 100 μg/ml ampicillin selecting for the pHUSEC41-1 plasmid) to determine the number of viable bacterial cells as well as the fraction containing the pHUSEC41-1 plasmid. CFU values were normalized per gram of tissue (CFU/g). The plasmid was maintained stably in all competitions and conjugational transfer between competing strains was assessed through replica-plating. The competitive index was calculated by dividing the output on days 2, 4, and 7 by the input on day 0.

### Ethics statement

Animal experiments were performed in accordance with national (regulation SJVFS 2012:26) and institutional guidelines. The Uppsala Animal Experiments Ethics Review Board in Uppsala, Sweden approved all mouse protocols undertaken in this study under reference no. 154/14. Animal experiments were performed at the Swedish National Veterinary Institute (SVA) in Uppsala, Sweden.

## Results and discussion

### Study material

**Figure 1:**
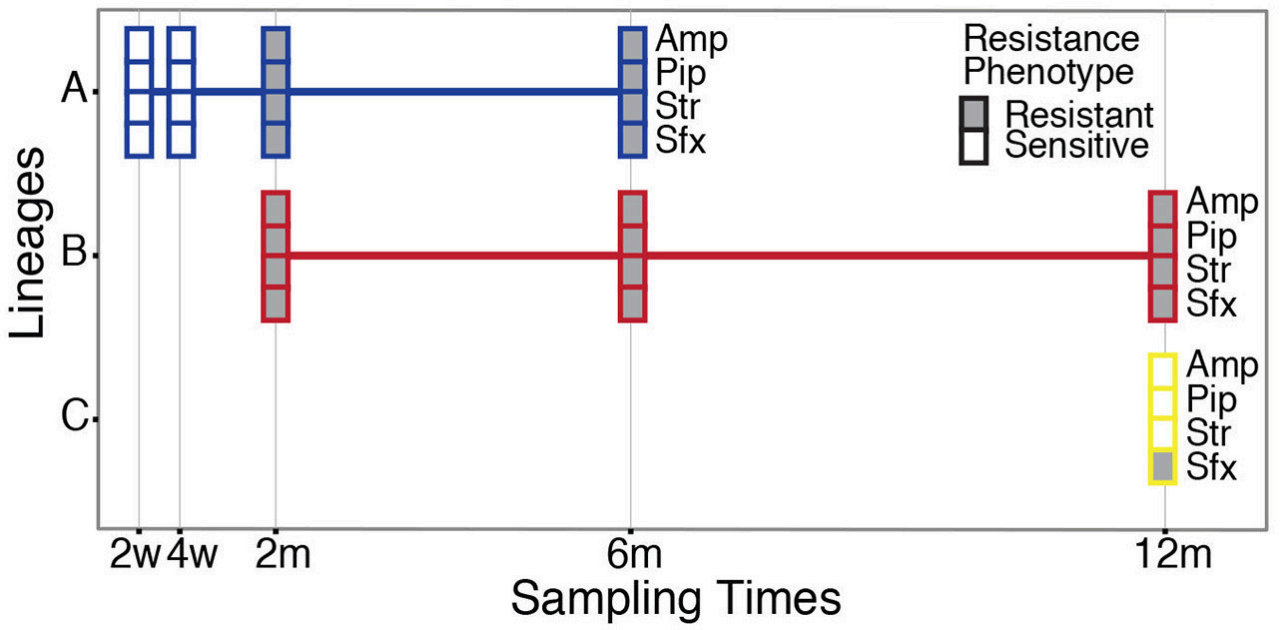
Sampling and antibiotic resistance profile of the *E. coli* isolates. A total of three *E. coli* lineages **(A–C)** were sampled from the infant's intestinal microbiota over the first year of life. Boxes indicate both the presence of the lineages and their antibiotic resistance profile to ampicillin (Amp), piperacillin (Pip), streptomycin (Str), and sulfamethoxazole (Sfx) at the sampling points. Filled, boxes indicate resistant isolates, and empty boxes indicate sensitive isolates (see Table S1 for MIC values).

**Figure 2:**
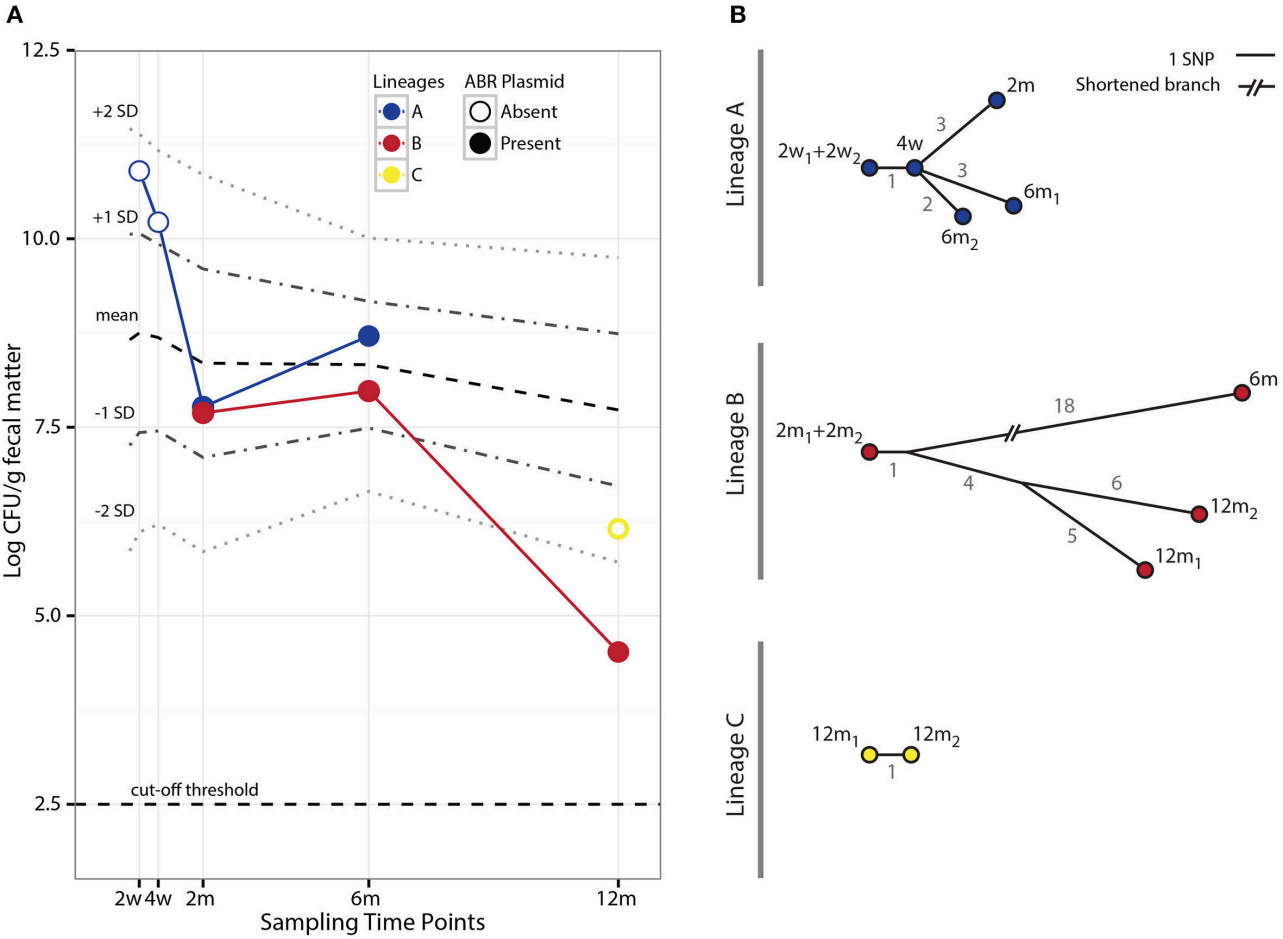
Population counts and SNP evolution of co-existing *E. coli* lineages. **(A)** Fecal population counts of *E. coli* lineages A, B, and C at different sampling points during the first year of life of the infant studied. Filled circles indicate the presence of the pHUSEC41-1-like antibiotic resistance plasmid. For comparison, the mean population levels and ±1 and 2 standard deviations (*SD*) at the same sampling points for 272 *E. coli* strains isolated from 128 infants in the ALLERGYFLORA cohort are indicated in the figure. **(B)** Phylogenetic trees based on the number of SNPs found in each of the isolates of lineages A and B. The gray values next to each branch indicates the number of SNPs between isolates.

Our study material was selected from an infant enrolled in the ALLERGYFLORA study, which was designed to examine the link between the infant gut microbial colonization pattern, over the first year of life, and the development of allergies (Nowrouzian et al., 2003). Fecal samples were cultured for *E. coli* and various colony types were assigned to specific lineages via random amplified polymorphic DNA and enumerated separately (RAPD; Figure 1). Sampling at 3 days and 1 week yielded no *E. coli* isolates. Between 2 weeks and 12 months a total of three distinct lineages were identified: A, B and C (Figure 1). The sampling at 2 and 4 weeks after birth yielded only colonies belonging to lineage A, which were sensitive to all antibiotics tested (Table S1). At the 2 month sampling time, lineage B appeared and resistance to the antibiotics: ampicillin, piperacillin, streptomycin, and sulfamethoxazole was measured. At this sampling time, the antibiotic resistance profile of lineage A changed, and subsequent isolates were now resistant to ampicillin, piperacillin, streptomycin, and sulfamethoxazole, matching the resistance profile of lineage B. lineages A and B were both present at the 6 months sampling time with no changes in the antibiotic resistance profile. At the 12 month sampling time, only lineage B remained, with the addition of lineage C, which was resistant to sulfamethoxazole. From plate-count estimations, we observed a consistent decrease in population numbers of *E. coli* in the gut of the infant over the first year of life (Figure 2A). This is in line with the other infants enrolled in the ALLERGYFLORA study and in parallel with the establishment of a microbiota dominated by anaerobic bacteria (Nowrouzian et al., 2003; Palmer et al., 2007).

### Genomic relationship of the lineages isolated from the gut

A total of 13 isolates from lineages A, B, and C were genome sequenced with at least one isolate sequenced per lineage per sampling point. Lineage A included two isolates from the 2 week sampling time (2w_1_ and 2w_2_), one from 4 weeks (4w) and 2 months (2m) and two from 6 months (6m_1_ and 6m_2_) lineage B included two isolates form 2 months (2m_1_ and 2m_2_) one from 6 months (6m) and two from 12 months (12m_1_ and 12m_2_) and lineage C isolates included two from 12 months (12m_1_ and 12m_2_). To confirm lineage identities of the isolates, we assessed both the number of SNPs and the amount of total genomic content shared between lineages by comparing to the first isolate sampled from each lineage.

Both lineages A and C had ~90,000 single nucleotide differences when compared to lineage B (Table S2). Interestingly, lineages A and C were less different with an order of magnitude fewer SNP when compared to each other; having ~7,000 SNPs. Similarly, when comparing the percentage of the genomic content shared between the lineages, lineages A and C shared between 79.7 and 82.3% in common with lineage B, whereas lineage A and C shared at least 93.6% of the genomic content (Table S3). While these results indicate that lineages A and C are more closely related to each other than to lineage B, the number of SNPs and the differences in genomic content reveal that they are different lineages. While RAPD-typing of the isolates was sensitive enough to successfully classify the isolates into the three distinct lineages, MLST typing assigned both lineage A and C isolates to ST12, whereas the lineage B isolates belonged to ST782.

Evolutionary relationships amongst the isolates within each lineage were established based on the SNPs identified by aligning reads to an isolate from the first time point the lineage was sampled (Table S4). SNPs identified in isolates from lineages A, B, and C produced consistent phylogenetic relations that show a progression in the acquisition of SNPs; indicating that the samples were representative clones of the lineages (Figure 2B).

### Multiple antibiotic resistance plasmid transfer *in situ* of the gut in the absence of antibiotic pressure

To identify the genomic changes underlying the acquisition of antibiotic resistance in lineage A, sequence data collected from the sensitive isolates (2w_1_, 2w_2_, and 4w) were compared to sequence data from the resistant isolates (2m, 6m_1_, and 6m_2_). Two non-conservative genomic mutations in the betaine aldehyde dehydrogenase (*betB*) and phosphoenolpyruvate carboxylase (*pckA*) genes were identified; however, these mutations would not be expected to contribute to antibiotic resistance. Instead, additional genetic information, totaling 90 kb, was found in the resistant lineage A isolates compared to sensitive lineage A isolates (Figure 1). The newly acquired genetic information had a read coverage two times greater than the chromosome, and included conjugative transfer genes; suggesting a newly acquired plasmid with ~2 copies per chromosome. Additionally, the following resistance genes were identified: the β-lactamase *bla*_TEM−1c_, an aminoglycoside 3′-phosphotransferase (*strA*), and streptomycin phosphotransferase (*strB*), as well as the dihydropteroate synthase gene (*sul2*), conferring resistance to sulfonamides.

The phenotypic resistance patterns (Figure 1) suggested that the horizontally acquired resistance was transferred from lineage B to lineage A. Aligning reads from lineage B to the newly acquired plasmid in lineage A resulted in 100% identity with only one identified SNP variant. Although we cannot out rule that the plasmid was already present in lineage A, or transferred from other constituents of the microbiota, the high degree of identity between the plasmids, the co-appearance of lineage B and a matching resistance profile is consistent with lineage B transferring its antibiotic resistance plasmid to lineage A.

**Figure 3:**
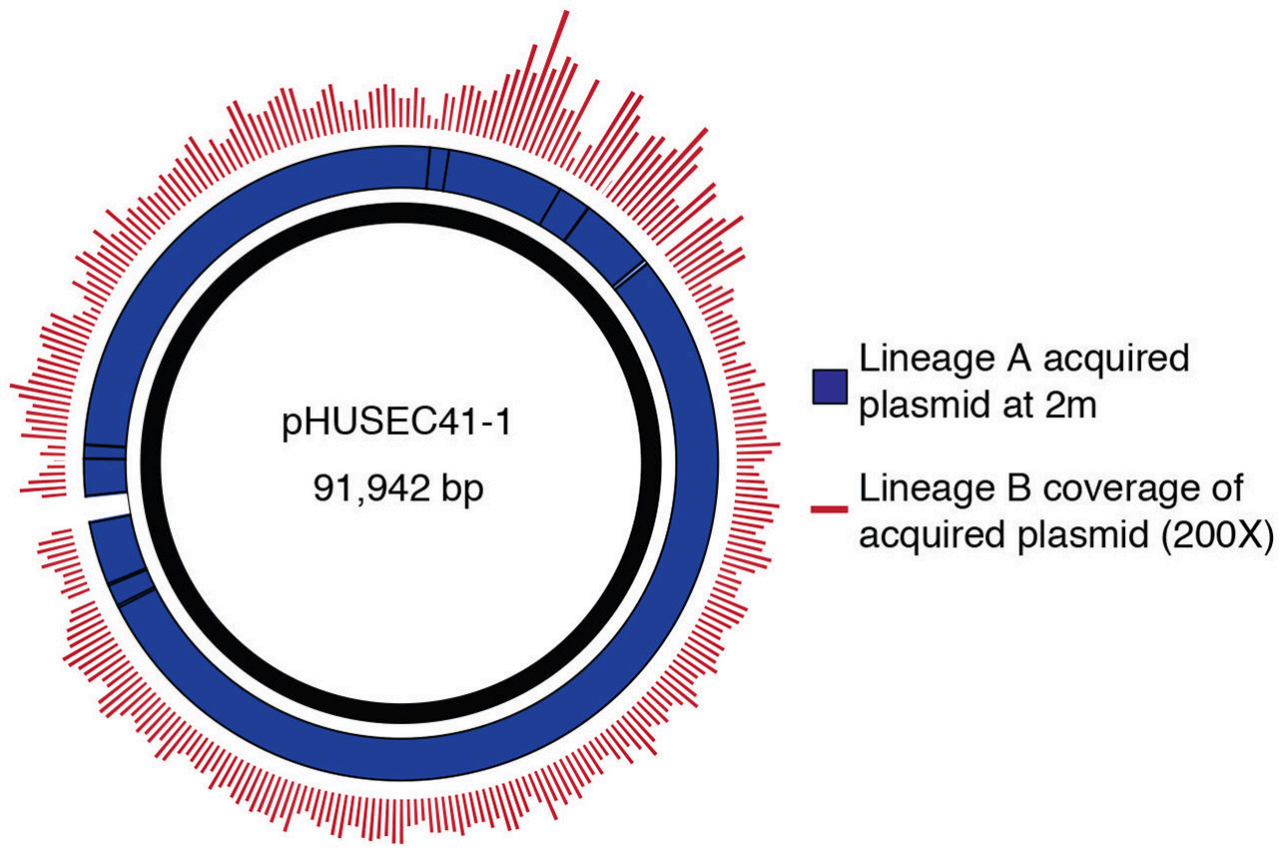
Transfer of a plasmid mediating antibiotic resistance. Contigs corresponding to the newly acquired plasmid were identified by analyzing differences in the read alignment coverage before and after the change in the resistance profile. Reads from lineage B are mapped to the acquired plasmid contigs of A, displaying coverage depth. High coverage and identity between the strains was observed. The acquired plasmid contigs of A were aligned to the sequence of pHUSEC41-1.

Querying sequence databases yielded the clinically important conjugative, IncI1-type pHUSEC41-1 plasmid of 91,942 bp (Grad et al., 2013). Contigs from the isolates in this study aligned to pHUSEC41-1 resulted in 99.3% coverage of the plasmid with an average of 99.0% identity (Figure 3). The alignment also showed that there were no insertions in the transferred plasmid compared to pHUSEC41-1. The pHUSEC41-1 plasmid was initially identified in the *E. coli* serotype O104:H4 strain HUSEC41 isolated from a child in Germany with hemolytic-uremic syndrome (HUS; Künne et al., 2012). This plasmid has additionally been found in other sequenced *E. coli* isolates of different serotypes isolated from patients in France and Finland (Grad et al., 2013); highlighting the wide dissemination of this multiple antibiotic resistance plasmid amongst geographically dispersed *E. coli* strains.

### The pHUSEC41-1-like resistance plasmid is costly *in vitro* but beneficial *in vivo* of the mouse gut

Interestingly, the acquisition of the pHUSEC41-1-like resistance plasmid by lineage A was associated with an initial steep drop in population counts, from 10^10.2^ CFU/g of fecal matter in the 4 week sample to 10^7.8^ CFU/g in the 2 month sample (Figure 2A). To determine whether this decrease related to a fitness cost imposed on lineage A from carrying the resistance plasmid, we conducted pair-wise *in vitro* competition experiments comparing the growth of a lineage A before and after the acquisition of the plasmid; namely lineage A 4w and 2m isolates. In these experiments, carriage of the plasmid incurred a cost of 6.3% (±1.9%) per generation on lineage A. However, despite the *in vitro* fitness cost of plasmid carriage, and the lack of obvious selection, the lineage persisted in the gut for at least another 4 months; showing an increase in cell counts during this time (Figure 2A).

**Figure 4:**
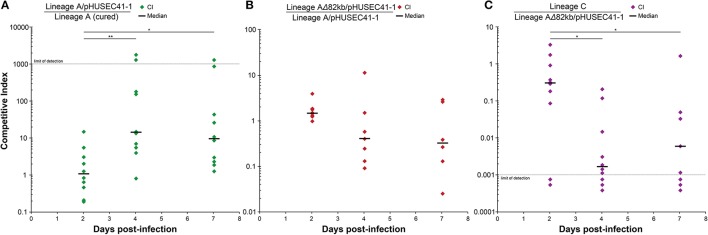
*In vivo* competition experiments. For each experiment, 10 mice were inoculated orally with equal amounts of two strains to quantify their relative fitness in a mouse gut environment. **(A)** The two lineage A isolates, with and without the pHUSEC41-1-like plasmid, were competed to assess the fitness effect of plasmid carriage *in vivo*. **(B)** The fitness effect of the 82kb deletion in lineage A, occurring between the 2 and 6 month time points, was hypothesized to be advantageous, but no significant fitness increase was measured for the deletion-isolate. **(C)** Lineage C was competed against the 6 months lineage A isolate to assess the potential role of lineage C in the disappearance of lineage A. Competitive indexes were analyzed relative to day 2 using the non-parametric Mann–Whitney *U*-test with a *P* < 0.05 considered significant and the degrees of statistical significance presented as ^**^P < 0.01 or ^*^P < 0.05.

We speculated that while the pHUSEC41-1-like plasmid slowed the growth of lineage A host *in vitro*, these conditions do not reflect the natural habitat of the strains and important environmental factors might contribute to fitness advantage of plasmid-carried genes *in vivo*. Therefore, to assess whether the plasmid provided a fitness advantage in a model gut environment, we tested the fitness of the plasmid bearing strain in the mouse gut before and after acquisition of the plasmid. Here we observed that the plasmid-carrying isolate out-competed the plasmid free isolate, and that the plasmid conferred a fitness advantage to lineage A in the mouse gut (*P* > 0.01; Figure 4A).

The fact that the lineage A transconjugant survived, increased its population counts, and exhibited a fitness advantage *in vivo*, highlights that resistance genes may more readily disseminate, and persist in healthy individuals never treated with antibiotics, than previously believed. However, studies examining the cost of plasmid carriage are often performed *in vitro*, and disagreement between *in vitro* and *in vivo* fitness measurements observed here, emphasizes the importance of investigating the persistence of antibiotic resistance in more natural settings.

While efforts have been devoted to studying the persistence of multidrug resistance plasmids in clinical *E. coli* isolates (Porse et al., 2016), our knowledge on the behavior of natural plasmids *in situ* of their native environment is limited (Conlan et al., 2014, 2016; Porse et al., 2017). While some studies show that stable inheritance and adaptive traits are crucial for long term plasmid survival (Simonsen, 2010), others suggest that certain conjugative plasmids can maintain themselves if present in their natural habitat of structured biofilms (Fox et al., 2008; Madsen et al., 2013). A substantial portion of pHUSEC41-1 encodes the *tra* genes involved in conjugative transfer. In addition to the effect of horizontal dissemination on plasmid persistence, conjugative transfer systems of plasmids have previously been shown to enhance adhesion and biofilm formation; features that may provide a survival advantage in the densely populated and structured environment of the gut (Ghigo, 2001; Fox et al., 2008; Madsen et al., 2013).

We tested the conjugative ability of the plasmid *in vitro* as well as *in vivo* of the mouse gut and found that the pHUSEC41-1-like plasmid conjugates at frequencies above 10^−6^ transconjugants per donor in all the tested conditions. In the mouse gut, an average of 10.8% of lineage A population had received the pHUSEC41-1-like plasmid from the lineage B strain at the final day 7 time point, indicating that the plasmid is actively conjugating in this environment.

In addition, pHUSEC41-1 encodes numerous proteins of unknown function that could potentially benefit its host *in vivo*, but further molecular analysis would be required to elucidate their role in plasmid persistence. However, candidate genes mediating the *in vivo* selection of pHUSEC41-1 could be factors involved in cobalamin biosynthesis (*cbiX*), DNA repair (*impCAB*; (Runyen-Janecky et al., 1999; Bali et al., 2014)), and conjugational transfer (*tra*). The CbiX protein can function as the terminal enzyme in siroheme biosynthesis in *E. coli*, which is known to aid iron utilization by its host (Bali et al., 2014). Iron is often restricted in the human body, and the ability to exploit these limited iron resources has been linked to increased persistence of *E. coli in vivo* (Andrews et al., 2003). pHUSEC-41-1 also harbors the *imp* operon, encoding an error-prone DNA repair system, that has been linked to increased survival following mutagenesis in a *Shigella* host and could similarly enhance the survival of *E. coli* hosts exposed to stressful conditions of the gut (Runyen-Janecky et al., 1999).

### A large deletion observed in lineage a was associated with an increase in population counts *in situ*

**Figure 5:**
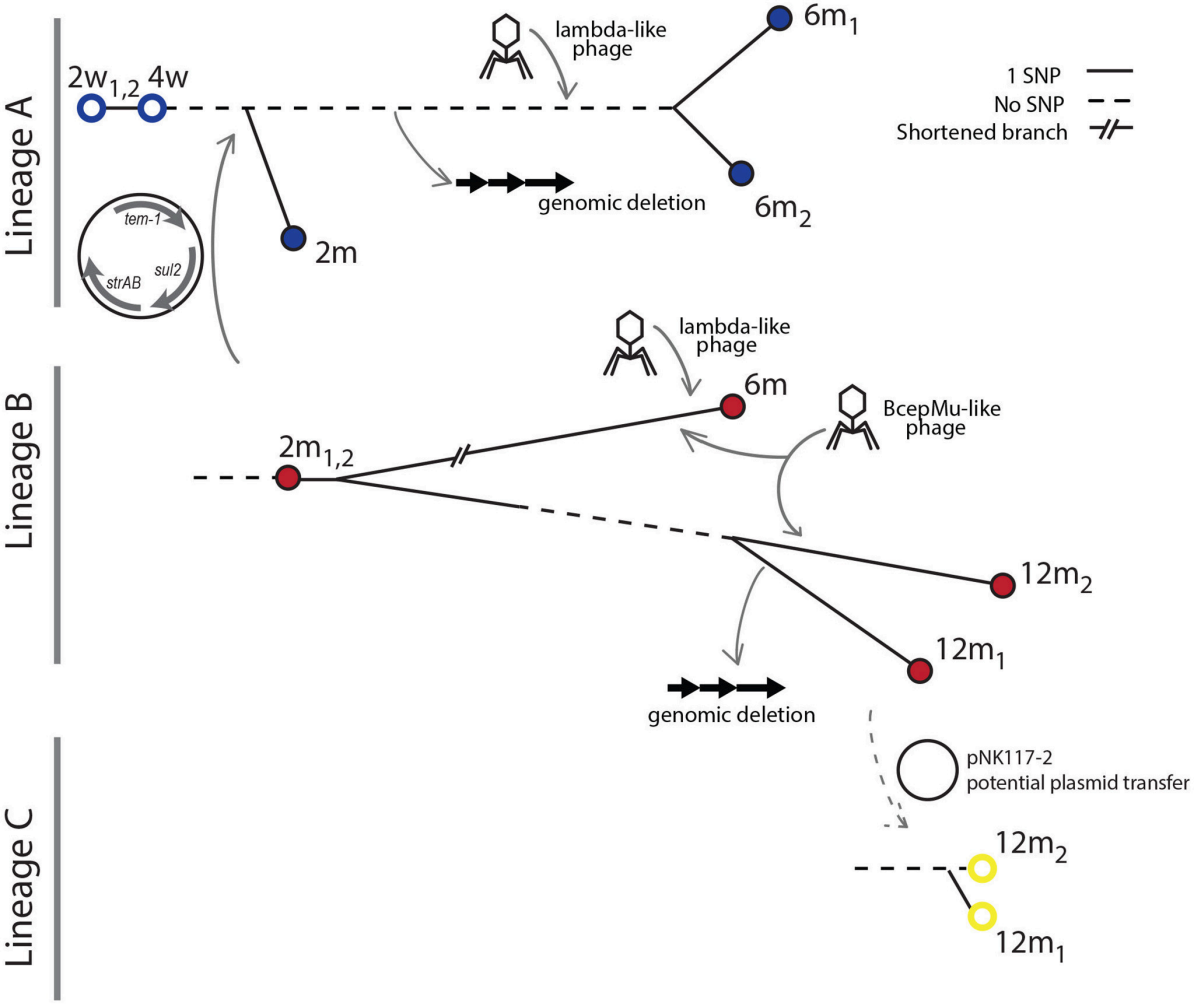
Overview of lineage genome dynamics. The transfer of a multidrug resistance plasmid from lineage B to lineage A occurred prior to the 2 month sampling time. The transfer occurred before diversification of the A lineage. At the 6 month sampling point, a Bcep-mu like phage infecting the B lineage was detected. In addition, both the A and B lineages were infected by lambda-like phages at this time point. A large genomic deletion occurred in the A lineage after the 2 month but before the 6 month sampling point. No isolates of lineage A were obtained at the final sampling time at 12 months, but a new isolate from lineage C is sampled along with lineage B.

After the acquisition of the pHUSEC41-1-like plasmid, a large deletion was detected in lineage A isolates at the 6-month sampling point (Figure 5). The deletion totaled 100.4 kb, aligned to a contiguous region in *E. coli* strain CFT 073 (NC_004431) and PCR assays confirmed the deletion (Figure S1). Annotated genes located in the region included iron scavenging genes, such as the *iroA* gene cluster and the hemolysin activator protein, peptide antibiotic genes microcin H47 and colicin-E1, which target *E. coli*, and antigen 43, which may have a role in adhesion (Cascales et al., 2007; Selkrig et al., 2012). Lastly, genes involved in fatty-acid synthesis, carbohydrate, and amino acid metabolism were also lost as a result of the deletion (See Table S5 for a complete list). A smaller chromosomal deletion was also identified in the lineage B 12m_1_ isolate (Figure 5). The deleted region totaled 26 kb and included genes characteristic for horizontally acquired DNA; including P fimbriae encoded by the *pap* genes as well as mobile element genes (See Table S6 for complete list).

At the 2 month sampling time, when lineage B was first sampled, lineages A and B had roughly the same population counts, at 10^7.8^ and 10^7.7^ CFU/g, respectively (Figure 2A). However, in contrast to lineage B, the population counts of lineage A increased by an order of magnitude at 6m. Upon receiving a foreign plasmid, antagonistic interactions between horizontally acquired chromosomal and plasmid factors might lower the fitness of the host e.g., due to overlapping gene or regulatory functions and these may be compensated through deletions (San Millan et al., 2015; Porse et al., 2016). To assess whether the large deletion, that occurred in lineage A between the 2 and 6 months sampling point, served as an adaptive response for lineage A, we performed an *in vivo* fitness assessment in the mouse gut between lineage A 2m and 6m, representing isolates before and after the large deletion. We did not find a statistically significant difference in fitness of the lineage A isolates with and without the large deletion (Figure 4B), suggesting that the deletion did not drive the increased population counts more than plasmid carriage in itself. As samples were not obtained between the 2m and 6m time points, and a Lambda-like phage infection also occurred within this time-span, a potential beneficial effect of the deletion could be outweighed by the subsequent phage acquisition. Just as acquisition of plasmid DNA can alter cell homeostasis, features of the acquired plasmid such as conjugation are known to induce the SOS response, which can increase the rate of genome rearrangements (Baharoglu and Mazel, 2014).

### Incoming lineage C shares an IncX plasmid with lineage B and establishes in the gut despite inferior fitness in *in vivo* experiments

Lineage C was sampled for the first time at the final 12m sampling time point. As the related lineage A was not sampled at this time and the counts of lineage B were the lowest sampled, we hypothesized that lineage C could be superior in terms of its ability to survive and compete in the gut. Therefore, we performed an *in vivo* fitness assessment in the mouse gut between the previously most abundant lineage A 6m and lineage C 12m strains. We found, in contrast to our hypothesis, that the lineage A 6m isolate out-competed the lineage C isolate in the mouse gut (*P* < 0.05; Figure 4C). Although the fitness of *E. coli* lineages is likely to vary between the human and mouse intestine, this result indicates that other factors of the complex gut environment not related to the appearance of lineage C, such as interactions with the remaining constituents of the microbiota or phage predators, may have played a more prominent role in the disappearance of lineage A. In addition, lineage C harbored a plasmid of 35.8 kbp, termed pNK117-2, which contained the *pilx* conjugation system similar to that of pOLA52 (Norman et al., 2008; Figure S2). Interestingly, pNK117-2 had 100% similarity to a plasmid from lineage B and might have been transferred from lineage B to lineage C. While we cannot demonstrate a second *in situ* transfer event, as we did not sample lineage C previously without pNK117-2, the presence of pNK117-2 in both lineage B and C with 100% sequence identity further exemplifies how plasmids can experience rapid dissemination in the absence of obvious selection.

## Conclusions

This work highlights the advantages of studying the longitudinal dynamics of co-existing bacterial lineages in the gut microbiota as a complement to metagenomic sequencing efforts. The power of this approach is expected to increase as cultivation methods for representative sampling of the gut microbiota improves further, and we anticipate that studies augmenting metagenomic sequencing with genomic sequencing and *in vivo* fitness models will provide a richer and more detailed view of the highly dynamic nature of individual genomes and HGT in the human gut microbiota. The substantial genome plasticity captured in this study highlights the dynamic nature of individual genomes of the gut microbiota. Of particular interest, we identify the transfer of a multi-drug resistance plasmid at the genomic level between co-existing bacterial lineages in the unperturbed human gut. Our findings suggest that, even though antibiotic resistance genes are not considered beneficial in the absence of antibiotic selection, they may hitchhike along with other selected traits. Further studies investigating the molecular mechanisms responsible for host compatibility and persistence of endemic antibiotic resistance plasmids *in situ* will refine our knowledge on the existence conditions of mobile elements, which will allow a better understanding of their role in the epidemiology and evolution of pathogenic bacteria.

## Author contributions

MS, HG, AP, DA, and JK conceived and designed the study. IA and AW designed the ALLERGYFLORA study and isolated the strains used in the present study. HG conducted the genomic analysis and strain phenotyping. NK performed the initial typing of the *E. coli* lineages, the phenotypic resistance testing, and the *in vitro* fitness cost assays. AP aided in strain sequencing, did *in vitro* conjugation assays, finalized the manuscript and tagged the isolates with resistance markers that JK and ML used to perform *in vivo* fitness experiments. HG, AP, and CM wrote the manuscript with input from JK, MS, ML, NK, IA, AW, and DA.

## Data availability

All sequenced genomes can be accessed via the Bioproject PRJNA396689.

### Conflict of interest statement

The authors declare that the research was conducted in the absence of any commercial or financial relationships that could be construed as a potential conflict of interest.

## Supplementary material

Figure S1 Large deletion in the genome of lineage A. Contigs from strain A were aligned to reference genome CFT073. Dark gray colored contigs represent regions flanking the excision. Pale colored contigs represent the region lost due to the deletion. Arrows indicate the position of the primers designed based on the CFT073 genome used to confirm the genomic excision.

Figure S2 Plasmid map of pHK117-2. Plasmid NK117-2 identified in both lineage B and C compared to IncX1 plasmids pRPEC180_47 (middle ring, blue) and pOLA52 (outer ring, green). Open reading frames (ORFs) identified on pNK117-2 are drawn in the inner most ring in black, with arrows indicating the reading direction. Annotations for selected ORFs are labeled outside of the rings.

Table S1 Antibiotic susceptibility tests of selected isolates. MIC values of lineages A, B, and C for sampling time points. One isolate per lineage per sampling time was selected for MIC testing.

Table S2 SNP comparison lineage A and B. Number of SNPs between the lineages using selected isolates. The rows of the table indicate the reads that were used aligned to contigs of the isolate as indicated in the column.

Table S3 SNP comparison lineage A and C. Coverage of the total genomic content between the lineages using selected isolates. The rows of the table indicate the reads that were used aligned to contigs of the isolate as indicated in the column.

Table S4 Within lineage SNPs. Table containing the SNPs from lineage A, B, and C, respectively, including the annotation and whether the amino acid change was synonymous or non-synonymous.

Table S5 Deleted genes from Linage A. List of annotated genes identified in the deleted chromosomal region of lineage A.

Table S6 Deleted genes from Linage B. List of annotated genes identified in the deleted chromosomal region of lineage B.
