# Supplementary figures and images for: Transfer and Persistence of a Multi-Drug Resistance Plasmid in situ of the Infant Gut Microbiota in the Absence of Antibiotic Treatment
Source: Front Microbiol. 2017 Sep 26;8:1852. doi: 10.3389/fmicb.2017.01852 (PMC5622998; doi:10.3389/fmicb.2017.01852)

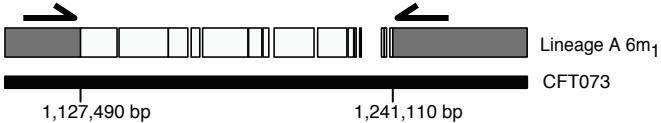

Supplement: Figure S1 — Large deletion in the genome of lineage A. Contigs from strain A were aligned to reference genome CFT073. Dark gray colored contigs represent regions flanking the excision. Pale colored contigs represent the region lost due to the deletion. Arrows indicate the position of the primers designed based on the CFT073 genome used to confirm the genomic excision. [file Image1.PDF]

pRPEC180\_47

100% identity

70% identity

50% identity

pOLA52

100% identity

70% identity

50% identity

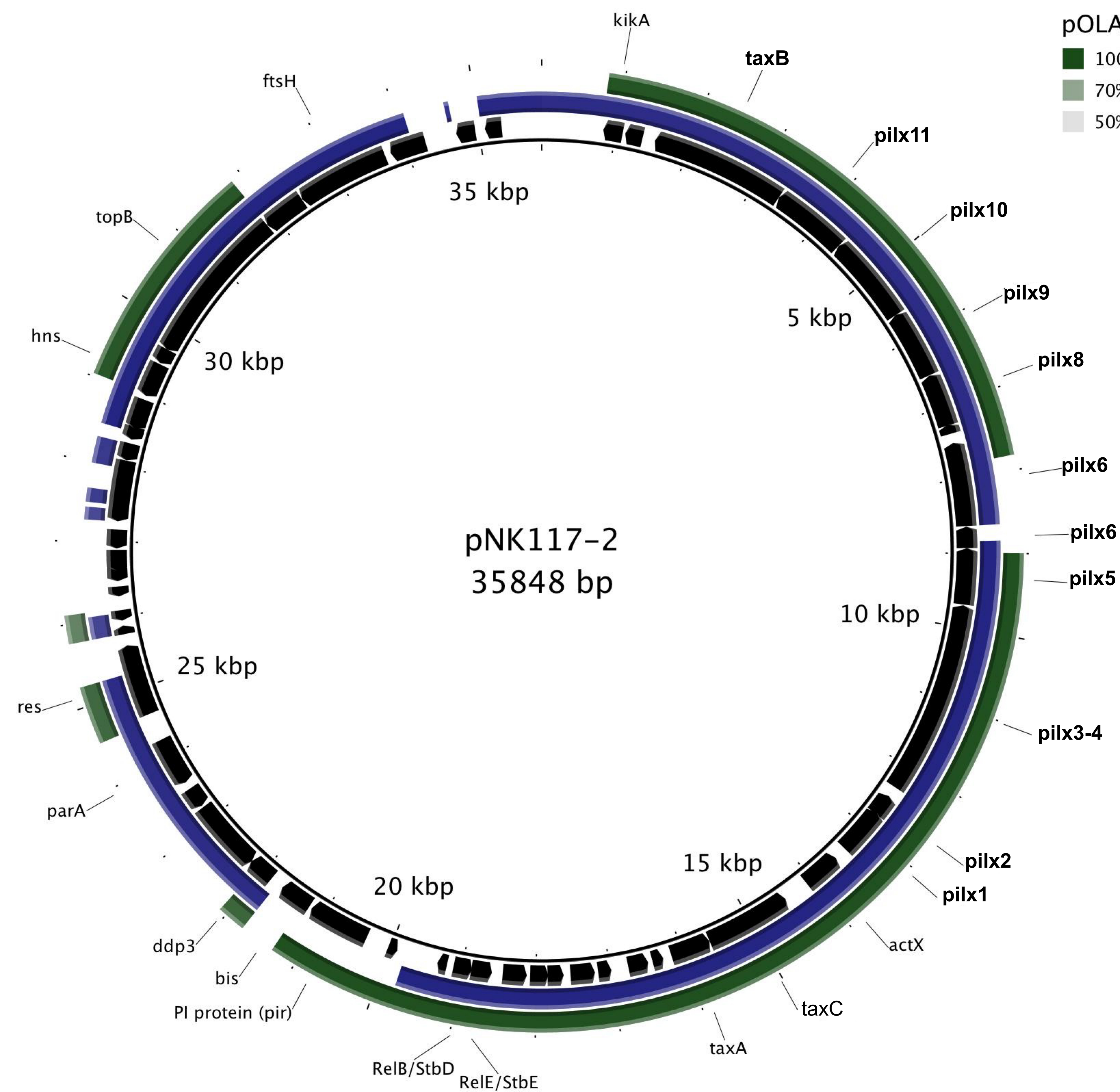

Supplement: Figure S2 — Plasmid map of pHK117-2. Plasmid NK117-2 identified in both lineage B and C compared to IncX1 plasmids pRPEC180_47 (middle ring, blue) and pOLA52 (outer ring, green). Open reading frames (ORFs) identified on pNK117-2 are drawn in the inner most ring in black, with arrows indicating the reading direction. Annotations for selected ORFs are labeled outside of the rings. [file Image2.PDF]
